# Trachoma and Relative Poverty: A Case-Control Study

**DOI:** 10.1371/journal.pntd.0004228

**Published:** 2015-11-23

**Authors:** Esmael Habtamu, Tariku Wondie, Sintayehu Aweke, Zerihun Tadesse, Mulat Zerihun, Zebideru Zewdie, Kelly Callahan, Paul M. Emerson, Hannah Kuper, Robin L. Bailey, David C. W. Mabey, Saul N. Rajak, Sarah Polack, Helen A. Weiss, Matthew J. Burton

**Affiliations:** 1 International Centre for Eye Health, London School of Hygiene and Tropical Medicine, London, United Kingdom; 2 The Carter Center, Addis Ababa, Ethiopia; 3 Amhara Regional Health Bureau, Bahirdar, Ethiopia; 4 The Carter Center, Atlanta, Georgia, United States of America; 5 International Trachoma Initiative, Atlanta, Georgia, United States of America; 6 International Centre for Evidence in Disability, London School of Hygiene and Tropical Medicine, London, United Kingdom; 7 Clinical Research Department, London School of Hygiene and Tropical Medicine, London, United Kingdom; 8 Department of Infectious Disease Epidemiology, London School of Hygiene & Tropical Medicine, London, United Kingdom; University of Tennessee, UNITED STATES

## Abstract

**Background:**

Trachoma is widely considered a disease of poverty. Although there are many epidemiological studies linking trachoma to factors normally associated with poverty, formal quantitative data linking trachoma to household economic poverty within endemic communities is very limited.

**Methodology/Principal Findings:**

Two hundred people with trachomatous trichiasis were recruited through community-based screening in Amhara Region, Ethiopia. These were individually matched by age and gender to 200 controls without trichiasis, selected randomly from the same sub-village as the case. Household economic poverty was measured through (a) A broad set of asset-based wealth indicators and relative household economic poverty determined by principal component analysis (PCA, (b) Self-rated wealth, and (c) Peer-rated wealth. Activity participation data were collected using a modified ‘Stylised Activity List’ developed for the World Bank’s Living Standards Measurement Survey. Trichiasis cases were more likely to belong to poorer households by all measures: asset-based analysis (OR = 2.79; 95%CI: 2.06–3.78; p<0.0001), self-rated wealth (OR, 4.41, 95%CI, 2.75–7.07; p<0.0001) and peer-rated wealth (OR, 8.22, 95% CI, 4.59–14.72; p<0.0001). Cases had less access to latrines (57% v 76.5%, p = <0.0001) and higher person-to-room density (4.0 v 3.31; P = 0.0204) than the controls. Compared to controls, cases were significantly less likely to participate in economically productive activities regardless of visual impairment and other health problems, more likely to report difficulty in performing activities and more likely to receive assistance in performing productive activities.

**Conclusions/Significance:**

This study demonstrated a strong association between trachomatous trichiasis and relative poverty, suggesting a bidirectional causative relationship possibly may exist between poverty and trachoma. Implementation of the full SAFE strategy in the context of general improvements might lead to a virtuous cycle of improving health and wealth. Trachoma is a good proxy of inequality within communities and it could be used to target and evaluate interventions for health and poverty alleviation.

## Author Summary

Trachoma has long been considered a disease of poverty. However, there is surprisingly little direct data that formally quantifies the relationship between trachoma and economic poverty, and none that specifically focuses on trichiasis. We compared 200 people with trachomatous trichiasis (TT) to 200 people (controls) without the condition, who were matched on age and sex, living in the same community, in Amhara Region, Ethiopia. We measured household relative poverty using three measures: household assets, self-rated wealth and peer-rated wealth. We also measured activity participation. We found TT case households were poorer by all relative economic measures. We found cases less likely to participate in economically productive activities regardless of visual impairment and other health problems, more likely to report difficulty and need assistance performing activities. The results suggest that the causative relationship between poverty and trachoma could possibly be bidirectional: poor households are more affected by trachoma and trichiasis reduces productivity even prior to development of visual impairment, which may exacerbate poverty. Implementation of the SAFE strategy in the context of general socioeconomic improvements might lead to a virtuous cycle of improving health and wealth. Trachoma could be used as proxy of inequality and to target and evaluate interventions for health and poverty alleviation.

## Introduction

Trachoma is leading infectious cause of blindness worldwide [1]. Trachomatous trichiasis (TT) is the late stage consequence of repeated conjunctival *Chlamydia trachomatis* infection in which eyelashes turn towards the eye, causing pain and eventually irreversible blinding corneal opacification (CO). About 229 million people live in trachoma endemic areas, and approximately 7.3 million have untreated TT [2,3]. More than 2.4 million people are visually impaired from trachoma worldwide, among which between 439,000 and 1.2 million are estimated to be irreversibly blind [2,4]. The WHO recommends the SAFE Strategy for trachoma control [5].This involves **S**urgery for trichiasis, **A**ntibiotics for infection, **F**acial cleanliness and **E**nvironmental improvements to suppress chlamydial infection and transmission.

Trachoma has long been considered a disease of poverty [6]. It is believed that the decline in trachoma observed in Europe, North America and elsewhere over the last century, in the absence of specific control measures, was largely attributable to general improvements in socio-economic status [7,8]. Trachoma remains prevalent in developing and marginalised communities, particularly in Africa, where crowded living conditions are common and access to clean water, sanitation and health care are often limited [6,8,9]. However, not all people living in such settings acquire active or scarring trachoma. It is possible that, within apparently homogeneous communities, the individuals who are most vulnerable to developing the blinding complications of trachoma are the poorest members of the poorest communities, although this has not been adequately investigated [10]. Moreover, the disability that TT causes may lead to reduced productivity, unemployment and loss of income, putting additional financial pressure on an already strained household [11–13]. The effect of trachoma on income may begin prior to the visual impairment, with the pain and the photophobia from trichiasis limiting function [13,14]. Of note, blindness has generally been associated with lower socio-economic status [15–17].

In low and middle income countries (LMICs) resources are often shared within households. Therefore, relative wealth or poverty in LMICs needs to be measured at household level, as the economic impact of a medical condition or intervention potentially affects the whole family [18]. In low-income settings estimating income can be difficult, as many people are self-employed and incomes are subject to significant short-term fluctuations [18,19]. In addition, people may earn from sources that they do not wish to disclose. Consumption expenditure data are considered more reliable than income data [16,19]. However, this method is subject to recall bias and requires detailed questionnaires, which are time consuming and costly to administer [19]. An alternative approach is to use a range of asset and housing characteristics as proxy indicators for household wealth and socio-economic status [19,20]. A key advantage of this approach is that it measures the long-term financial status of a household, and is less vulnerable to short-term fluctuations than income and consumption expenditure [19,20]. On the other hand, asset score only measure relative poverty, which may preclude regional or international comparability.

There is surprisingly little direct data that formally quantifies the relationship between trachoma and economic poverty, and none that specifically focuses on the scarring sequelae. The aim of this study was to investigate in detail the relationship between poverty and trachomatous trichiasis through an asset-based analysis, self-rated and peer-rated wealth measures, and participation in productive activities.

## Methods

### Ethics Statement

This study was reviewed and approved by the Food, Medicine and Healthcare Administration and Control Authority of Ethiopia, the National Health Research Ethics Review Committee of the Ethiopian Ministry of Science and Technology, Amhara Regional Health Bureau Research Ethics Review Board Committee, the London School of Hygiene and Tropical Medicine (LSHTM) Ethics Committee, and Emory University Institutional Review Board. Written informed consent in Amharic was obtained prior to enrolment from participants. If the participant was unable to read and write, the information sheet and consent form were read to them and their consent recorded by thumbprint.

### Study Design and Participants

This case-control study was nested within a clinical trial of two alternative surgical treatments for trichiasis. From the 1000 trichiasis cases recruited into the trial, every fifth consecutive case was also enrolled into this economic poverty study and matched to a non-trichiasis control. This approach was chosen for logistical and methodological reasons, in order to identify and collect data from controls within the shortest possible time period following case recruitment. Cases were defined as individuals with one or more eyelashes touching the eyeball or with evidence of epilation in either or both eyes in association with tarsal conjunctival scarring. People with trichiasis of other causes, recurrent trichiasis and those under 18 years were excluded. Trichiasis cases were identified mainly through community-based screening. Trichiasis screeners and counsellors (Eye Ambassadors) visited every household in their target village, identified and referred trichiasis cases to health facilities where surgical services were provided. Some individuals self-presented or were referred by local health workers. Recruitment was mainly from three districts of West Gojam Zone, Amhara Region, Ethiopia between February and May 2014. This area has one of the highest burdens of trachoma worldwide [21].

Controls were individuals without clinical evidence or a history of trichiasis (including surgery and epilation), and who came from households without a family member with trichiasis or a history of trichiasis, as we wanted to measure household level relative poverty, which requires comparison of trichiasis case households with households without trichiasis cases. One control was individually matched to each trichiasis case by location, sex and age (+/- two years). The research team visited the sub-village (30–50 households) of the trichiasis case requiring a matched control. A list of all potentially eligible people living in the sub-village of was compiled with the help of the sub-village administrator. One person was randomly selected from this list using a lottery method, given details of the study and invited to participate if eligible. If a selected individual refused or was ineligible, another was randomly selected from the list. When eligible controls were not identified within the sub-village of the case, recruitment was done in the nearest neighbouring sub-village, using the same procedures.

### Data Collection

Data on detailed demographic characteristics were collected. Household economic poverty was measured through (a) Asset based wealth indicators, (b) Self-rated wealth, and (c) Peer-rated wealth. Activity participation data was collected using a modified ‘Stylised Activity List’ developed for the World Bank’s Living Standards Measurement Survey [22]. Visual acuity of both cases and controls were measured and cases underwent detailed trachoma examination.

#### Asset-based wealth inequality indicators

Data on 60 asset variables were collected. This included (i) housing characteristics and utilities, (ii) ownership of durable assets, and (iii) ownership of agricultural assets. Most data were collected through direct observation. Data on access to water was not collected as it is mainly supplied by government and non-governmental organisations, therefore would not directly reflect the household’s wealth but rather general infrastructure development in the area. Households were asked about their financial savings and whether they had loans from the government at the time of data collection.

#### Self and peer-rated wealth indexes

The participants were asked the question: “*How well-off do you think your household is in relation to the other households in the village*?” They were then asked to choose one of the following options: (1) very poor, (2) poor, (3) average, (4) wealthy or (5) very wealthy. Three members of the village administration team (peers of both the cases and the controls) were then randomly selected and independently asked the question: *“How well-off do you think [Name of household head] household is in relation to the other households in the village*?*”* for both the case and the control households. They were asked to choose one of the five levels.

#### Activity participation data

The ‘Stylised Activity List’ tool contains a list of common activities in different subgroups: household activities, paid work, work for own use, leisure activities and personal activities.[22,23] Participants were asked if they had participated in any of the activities in the subgroups in the last week. If they had undertaken a specific activity in the last week, they were asked the question *“How much difficulty did you have in doing [Activity] in the last week*?*”* and asked to choose one of the following options: (0) extreme/not able to do, (1) a lot of difficulty, (2) some difficulty, (3) little difficulty, (4) no difficulty; and another question whether they have done the activity (1) fully assisted, (2) with some assistance, (3) with no assistance.

#### Visual acuity and clinical examination

Presenting LogMAR (Logarithm of the Minimum Angle of Resolution) visual acuity at two metres was measured using “PeekAcuity” software on a smartphone in a dark room [24]. The ophthalmic examination was conducted in a dark room using a 2.5x binocular magnifying loupe and a bright torch. Clinical signs were graded using the Detailed WHO Follicles Papillae Cicatricae (FPC) Grading System [25].

### Sample Size

To detect a difference in asset-based principal component analysis (PCA) similar to that found in the Cataract Impact Study (mean and standard deviation of asset based PCA score in cataract cases and their controls 0.6 and 2.0; and 0.3 and 2.6, respectively) with an alpha of 0.05 and 95% power, at least 346 (173 in each group) participants were required [16]. We recruited 200 trichiasis cases and 200 age, sex and location matched non-trichiasis controls.

### Analysis

Data were double-entered into Access (Microsoft) and transferred to Stata 11 (StataCorp) for analysis. Conditional logistic regression was used to compare basic characteristics of matched cases and controls.

#### Asset index analysis

Descriptive and summary statistics of all asset indicators were calculated. A PCA was used to analyse the asset-based wealth or inequality indicator data in order to classify households into different socio-economic levels [19,20,26–28]. Variables owned by less than 5% or more than 95% of the participants’ households were excluded from the PCA as they would have the least weight and less value in differentiating socio-economic status or inequality. The PCA was conducted separately to generate a factor score for each of the three subset asset indices: (1) housing and utilities, (2) durable assets and (3) agricultural assets, and for all asset variables combined [19,20,27]. The control households were grouped into quintiles based on the overall asset index socio-economic score (SES). Then the case households were classified based on the “cut points” of the controls’ socio-economic quintiles. We performed matched univariable and multivariable conditional logistic regression analyses to investigate the relationship between asset-based household economic poverty and case-control status. A stratified analysis was performed to test whether the observed association persisted in different groups. Logistic regression adjusted for clustering using robust standard errors was used for stratified analyses of all economic poverty measures by age, sex, marital status and vision, and variables of insufficient frequencies (such as government loan) for matched analysis. Likelihood ratio tests were used to obtain p-values in categorical exposure variables. To test for robustness of the asset index a Spearman rank correlation coefficient was employed to examine whether the three sub-set asset indices produce similar classifications of SES to the overall asset index. To adjust for multiple comparisons, we used the Benjamini and Hochberg method, assuming a false discovery rate (FDR) of 5% [29].

#### Self and peer-rated wealth indexes

The wealth scores provided by the three peers were averaged. The association between self and peer-rating of household socio-economic status and case-control status was examined using conditional logistic regression. The self-rated and peer-rated wealth scores were converted into a score out of one hundred, using the formula: ([individual score–lowest possible score]/[Highest possible score—lowest possible score])x100. Lower scores indicate a worse score (0 the lowest possible score) and higher scores indicates better score (100 the highest score) [30]. The mean scores were compared between cases and controls using the Wilcoxon rank-sum test. The correlations of the self-rated wealth, the peer-rated wealth and the asset index based socio-economic classifications of households were compared using Spearman rank correlation coefficient.

#### Activity and participation data

Activities undertaken (paid employment and commission work) and not undertaken (talking with friends) by <1% participants were excluded from the analysis. Activities were regrouped into productive household activities, outdoor activities, paid work, agricultural activities and leisure activities. The association between participation in an activity and case-control status were analysed using conditional logistic regression adjusting for self-reported health problems occurring in the last month. Logistic regression adjusted for clustering (using robust standard errors), age, sex and self-reported health problems was used to analyse the difference in activity participation between cases and controls stratified by vision, and to analyse the association between case-control status and difficulty in doing an activity and receiving assistance.

#### Clinical data

Presenting visual acuity in the better eye was used in analysis. For visual acuities of counting fingers or less, LogMAR values were attributed as follows: counting fingers, 2.0; hand movements, 2.5; perception of light, 3.0; and no perception of light, 3.5 [31]. The LogMAR visual acuity scores were categorised using the WHO classification: normal vision, ≥6/18; moderate visual impairment, <6/18 to ≥6/60; severe visual impairment, <6/60 to ≥3/60; and blind, <3/60. Corneal opacity grading and trichiasis grading in the more affected eye was undertaken to test their association with household economic poverty among trichiasis cases. Based on their severity, trichiasis cases were categorised into Minor Trichiasis cases with <6 lashes or evidence of epilation in <1/3^rd^ of the lash margin; and Major Trichiasis cases with ≥6 lashes or evidence of epilation in ≥1/3^rd^ of the lash margin.

## Results

### Demographic and Clinical Characteristics of Participants

Cases and controls were well matched in terms of location, gender and age and had similar levels of literacy, household size and household occupation (Table 1). Compared to the controls, the trichiasis cases were less likely to be married, more likely to be either unemployed or work as daily labourers, less likely to have a family member with formal education and more likely to have experienced a health problem during the last month. As expected, cases were more likely to be visually impaired than the controls (37.0% v 3.0%, respectively; OR = 69.0; 95%CI 9.58–496.82; p<0.0001)

**Table 1:** Demographic and clinical characteristics of individual participants and their households.

Variables	Cases		Controls		P-value
	n / 200	(%)	n / 200	(%)	
**Individual**					
***Age (years)*, *mean (SD)***	46.1	(13.5)	45.9	(13.3)	
***Gender*, *female***	167	(83.5)	167	(83.5)	
***Illiterate***	177	(85.5)	170	(85.0)	0.25
***Marital status***					
Married	130	(65.0)	162	(81.0)	0.0001^‡^
Widowed	38	(19.0)	27	(13.5)	
Divorced	27	(13.5)	9	(4.5)	
Single	5	(2.5)	2	(1.0)	
***Job***					
Farmer	158	(79.0)	168	(84.0)	0.006^‡^
Employed/self employed	9	(4.5)	17	(8.5)	
Daily labourer	14	(7.0)	4	(2.0)	
No job	19	(9.5)	11	(5.5)	
***Visual acuity–better eye***					
Normal (≥6/18)	126	(63.0)	194	(97.0)	<0.0001^†^
Moderate visual impairment (<6/18 to ≥6/60)	65	(32.5)	4	(2.0)	
Severe visual impairment (<6/60 to ≥3/60)	5	(2.5)	1	(0.5)	
Blind (<3/60)	4	(2)	1	(0.5)	
***Self reported health problem in the last month***					
No	115	(57.5)	172	(86.0)	<0.0001
Yes	85	(42.5)	28	(14.0)	
**Household**					
***Family size*, *mean (SD)***	4.9	(2.4)	5.1	(2.0)	0.17
***Highest family education***					
No formal education	41	(20.5)	22	(11.0)	0.006^†^
Primary school	74	(37.0)	74	(37.0)	
Secondary/high school	70	(35.0)	82	(41.0)	
Higher education	15	(7.5)	22	(11.0)	
***Highest family job***					
Farmer	165	(82.5)	169	(84.5)	0.29^‡^
Employed/self employed	18	(9.0)	21	(10.5)	
Daily labourer	16	(8.0)	9	(4.5)	
No job	1	(0.5)	1	(0.5)	

Analysis is done by conditional logistic regression.

^***‡***^ Combined p-value from likelihood ratio-test^.^

^†^ P-value for trend.

### Distribution of Assets

The asset variables used in the PCA are described in Table 2 and their summary statistics are shown in S1 Table. The PCA was based on a combination of 28 asset values. The other 32 measured assets were excluded as they were present in less than 5% or more than 95% of the participants’ households. Households were generally poor. About 67% had a latrine, among which 65% were of the “non-improved” pit latrine type without a concrete slab. About half (54%) had their cattle dwelling within the main house. Ownership of durable assets such as mobile phones and radio was low (<30%). Only 17% of the households had access to electricity. About 12% of the households had taken a government loan. Overall, cases had fewer household and agricultural assets than controls and were more likely to have a government loan (Table 2). There was no difference in the ownership of the house they were living in (92.0% vs 94%, p = 0.22), or access to electricity (18·5% v 16·5%, p = 0·40). Case households had fewer rooms (1.22 vs 1.55, p<0·0001), and had a higher density of persons per room than the controls: 4.0, 95%CI 3.6–4.4 vs 3.3, 95%CI 3.0–3.6 respectively (P = 0.020).

**Table 2:** Descriptive and summary statistics for all 28 asset variables that were included in the principal component analysis.

Variables	Cases (200)		Controls (200)		P-values*
	n or mean	(% or S.D.)	n or mean	(% or S.D.)	
***Housing characteristics and utilities***					
Own current house	184	(92.0%)	188	(94.0%)	0.22
Number of rooms, mean (SD)	1.22	(S.D. 0.61)	1.55	(S.D. 0.08)	<0.0001
Roof made of metal	175	(87.5%)	195	(97.5%)	0.0010
Number of metal roof sheets	41.3	(24.3%)	59.8	(29.3%)	<0.0001
Own other houses	16	(8.0%)	19	(9.5%)	0.58
Latrine availability	114	(57.0%)	153	(76.5%)	<0.0001
Separate kitchen area	56	(28.0%)	96	(48.0%)	<0.0001
Cattle dwelling within main house	117	(58.5%)	99	(49.5%)	0.04
Cattle dwelling outside main house	30	(15.0%)	66	(33.0%)	<0.0001
Access to Electricity	33	(16.5%)	37	(18.5%)	0.40
***Ownership of durable household materials***					
Phone	38	(19.0%)	59	(29.5%)	0.005
Radio	44	(22.0%)	72	(36.0%)	0.003
Number of household furniture, mean (SD)	0.99	(S.D. 0.73)	1.49	(S.D. 0.97)	<0.0001
Cart	6	(3.0%)	21	(10.5%)	0.002
***Agricultural assets (plants*, *land*, *animals)***					
Mango Trees	10	(5.0%)	21	(10.5%)	0.03
Guava Trees	7	(3.5%)	18	(9.0%)	0.02
Lemon Trees	10	(5.0%)	23	(11.5%)	0.02
Banana trees	12	(6.0%)	24	(12.0%)	0.04
Buckthorn trees	123	(61.5%)	151	(75.5%)	0.0004
Coffee land	10	(5.0%)	27	(13.5%)	0.004
Equaliptous land	79	(39.5%)	135	(67.5%)	<0.0001
Teff land in Hectares, mean (SD)	0.81	(S.D. 0.63)	1.11	(S.D. 0.77)	<0.0001
All lands in Hectares, mean (SD)	0.88	(S.D. 0.66)	1.19	(S.D. 0.80)	<0.0001
Animal Ownership					
Cattle, mean (SD)	2.76	(S.D. 3.06)	4.46	(S.D. 3.25)	<0.0001
Sheep/Goat, mean (SD)	1.24	(S.D. 2.23)	2.11	(S.D. 2.71)	<0.0002
Horse/mule/donkey, mean (SD)	0.35	(S.D. 0.73)	0.74	(S.D. 0.82	<0.0001
Chicken, mean (SD)	2.32	(S.D. 4.02)	3.46	(S.D. 4.43)	0.0065
***Government loan***	49	(24.5%)	1	(0.5%)	<0.0001^‡^

^*^ All p-values were derived from conditional logistic regression, with the exception of those for government loan‡, which used logistic regression models adjusted for clustering using robust standard errors method. Using the Benjamini and Hochberg method, only tests with a p-value below 0·0387 have a False Discovery Rate of <5%.

### Asset Index Factor Scores

**Fig 1:**
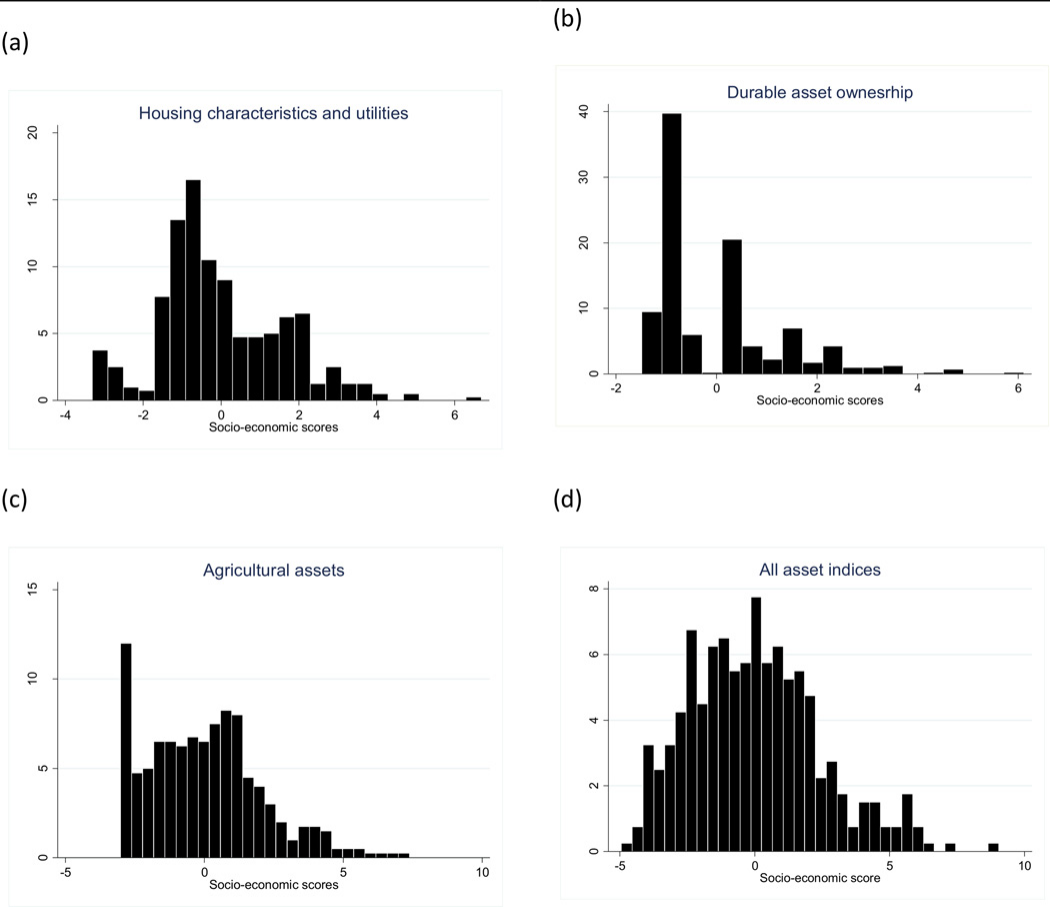
Distribution of socio-economic scores for (a) housing characteristics and utilities, (b) durable assets, (c) agricultural assets and (d) all assets combined.

The overall asset index accounts for 21% of the total variance (S1 Table). Among the three subset asset indices, the agricultural asset indicators had the highest factor scores and accounted for the highest weights in measuring wealth in this population. In contrast, the housing characteristics and utilities index, except for the number of metal roof sheets, had generally lower factor scores and contributed lower weights in estimating wealth than the other two subset indices. Among all indices, number of oxen and cows owned (0.324), the number of metal roof sheets (0.320) and amount of land owned in hectares (0.319) had the highest weights in estimating wealth. In contrast, access to electricity (-0.096) having cattle dwelling within the main house (-0.024) and having a government loan (-0.038) had negative weights. Fig 1 illustrates the distribution of the subset and overall asset indices, in order to determine whether clumping or truncation were present in this data. Overall, there was evidence of truncation and clumping when the three subset indices (Fig 1A to 1C) are used separately. However, the distribution of the overall combined factor scores was much smoother; and clumping and truncation were not observed (Fig 1D).

### Asset Based Household Economic Poverty and Trichiasis

There was a strong association between being a trichiasis case and asset based household economic poverty: OR = 2.79; 95%CI, 2.06–3.78; p<0.0001 (Table 3). This relationship persisted after adjusting for marital status, and highest family education (OR = 2.78; 95%CI, 2.00–3.87; p<0.0001). For stratified analyses we combined “richest” and “rich” with “middle” because of small numbers, to create a “middle & above” category with three levels of socio-economic status measure to facilitate data modelling. Compared to the controls, trichiasis cases were more likely to be from the poorest (OR = 2.65; 95%CI, 2.05–3.42; p<0.0001) households than from the middle & above households (Table 4). In the stratified analysis, the association between asset based household economic poverty and trichiasis persisted regardless of age, gender, marital status, and in people with normal visual acuity after adjusting for the matching variables and family education (Table 4).

**Table 3:** Association between household economic poverty and trachomatous trichiasis.

Poverty Index	Cases (200)		Controls (200)		Univariable analysis			Adjusted analysis ^a^		
	n	(%)	n	(%)	OR	(95% CI)	P-value	OR	(95% CI)	P-value
***Overall asset index*** ^†^										
Richest	9	(4.5)	40	(20.0)	2.79	(2.06–3.78)	<0.0001	2.78	(2.00–3.87)	<0.0001
Rich	20	(10.0)	40	(20.0)						
Middle	17	(8.5)	40	(20.0)						
Poor	51	(25.5)	40	(20.0)						
Poorest	103	(51.5)	40	(20.0)						
***Self-rated wealth index*** ^‡^										
Very wealthy	1	(0.5)	1	(0.5)	4.41	(2.75–7.07)	<0.0001	3.99	(2.43–6.54)	<0.0001
Wealthy	4	(2.0)	29	(14.5)						
Average	95	(47.5)	135	(67.5)						
Poor	67	(33.5)	32	(16.0)						
Very poor	33	(16.5)	3	(1.5)						
***Peer-rated wealth index*** ^‡^										
Very wealthy	1	(0.5)	5	(2.5)	8.22	(4.59–14.72)	<0.0001	9.10	(4.79–17.270)	<0.0001
Wealthy	8	(4.0)	35	(17.5)						
Average	48	(24.0)	124	(62.0)						
Poor	80	(40.0)	30	(15.0)						
Very poor	63	(31.5)	6	(3.0)						

Socio-economic classification of cases and controls into quintiles based on the first principal component factor scores of the overall asset index and; self and peers ranking of households’ wealth. Analysis is done using conditional logistic regression for trend after likelihood ratio-test for non-linearity.

^a^ Marital status and highest family education included in the matched analysis model.

^*†*^ The case households were classified based on the “cut points” of the controls’ socio economic quintiles.

^‡^ Socioeconomic classification households as rated by the study participants and their peers.

**Table 4:** The relationship between household economic poverty and trachomatous trichiasis using the asset index, self-rated wealth index and peer-rated wealth index, stratified by age, sex, marital status and vision.

Category	Asset Index					Self Rated Wealth Index					Peer Rated Wealth Index				
	Cases	Controls	OR	(95% CI)	P-value	Cases	Controls	OR	(95% CI)	P-value	Cases	Controls	OR	(95% CI)	P-value
**All (n = 400)**	**200**	**200**				**200**	**200**				**200**	**200**			
Middle & Above	23.0%	60.0%	2.7	(2.05–3.42)	<0.0001	50.0%	82.5%	3.7	(2.55–5.49)	<0.0001	28.5%	82.0%	10.6	(6.4–17.4)	<0.0001
Poor	25.5%	20.0%				33.5%	16.0%				40.0%	15.0%			
Poorest	51.5%	20.0%				16.5%	1.5%				31.5%	3.0%			
**Age** ^a^ ^‡^															
**Young (n = 200)**	**104**	**96**				**104**	**96**				**104**	**96**			
Middle & Above	24.0%	58.3%	2.6	(1.83–3.70)	<0.0001	61.5%	89.6%	3.4	(1.86–6.27)	0.0001	39.4%	87.5%	9.4	(4.63–19.3)	<0.0001
Poor	26.9%	21.9%				25.0%	9.4%				33.7%	11.5%			
Poorest	49.0%	19.8%				13.5%	1.0%				26.9%	1.0%			
**Old (n = 200)**	**96**	**104**				**96**	**104**				**96**	**104**			
Low	21.5%	61.9%	3.3	(2.17–5.10)	<0.0001	37.5%	76.0%	4.1	(2.46–6.99)	0.0001	16.7%	76.9%	13.6	(6.39–29.0)	<0.0001
Medium	24.0%	18.3%				42.7%	22.1%				46.9%	18.3%			
High	54.2%	20.2%				19.8%	1.9%				36.5%	4.8%			
**Sex** ^b^															
**Male (n = 66)**	**33**	**33**				**33**	**33**				**33**	**33**			
Middle & Above	36.4%	75.8%	4.4	(2.04–9.49)	0.0002	48.5%	84.9%	5.7	(1.68–19.2)	0.0053	30.3%	93.9%	27.3	(5.13–145)	0.0001
Poor	33.3%	21.2%				51.5%	15.1%				54.5%	6.1%			
Poorest	30.3%	3.0%				0.0%	0.0%				15.2%	0.0%			
**Female (n = 334)**	**167**	**167**				**167**	**167**				**167**	**167**			
Middle & Above	20.4%	56.9%	2.5	(1.85–3.25)	<0.0001	50.3%	82.0%	3.56	(2.38–5.32)	<0.0001	28.1%	79.6%	9.3	(5.54–15.7)	<0.0001
Poor	33.9%	19.8%				29.9%	16.2%				37.1%	16.8%			
Poorest	55.7%	23.3%				19.8%	1.8%				34.7%	3.6%			
**Marital status** ^c^															
**Married (n = 292)**	**130**	**162**				**130**	**162**				**130**	**162**			
Middle & Above	33.1%	67.9%	2.9	(2.10–3.96)	<0.0001	64.6%	88.9%	4.5	(2.63–7.55)	<0.0001	40.8%	90.7%	13.1	(6.66–25.8)	<0.0001
Poor	33.8%	20.4%				28.5%	11.1%				44.6%	8.6%			
Poorest	33.1%	11.7%				6.9%	0.0%				14.6%	0.6%			
**Unmarried** * **(n = 108)**	**70**	**38**				**70**	**38**				**70**	**38**			
Middle & Above	4.3%	26.3%	3.1	(1.57–6.01)	0.0011	22.9%	55.3%	3.1	(1.65–5.91)	0.0005	5.7%	44.7%	6.27	(2.81–14.0)	<0.0001
Poor	10.0%	18.4%				42.9%	36.8%				31.4%	42.1%			
Poorest	85.7%	55.3%				34.3%	7.9%				62.9%	13.2%			
**Visual acuity** ^a^															
**Normal (n = 320)**	**126**	**194**				**126**	**194**				**126**	**194**			
Middle & Above	27.0%	61.3%	2.5	(1.91–3.66)	<0.0001	57.1%	84.0%	4.0	(2.49–6.33)	<0.0001	32.5%	83.5%	11.1	(6.00–20.6)	<0.0001
Poor	26.2%	19.6%				30.2%	15.0%				41.3%	13.9%			
Poorest	46.8%	19.1%				12.7%	1.0%				26.2%	2.6%			
**VI (n = 80)** ^†^	**74**	**6**				**74**	**6**				**74**	**6**			
Middle & Above	16.2%	16.7%	5.0	(0.28–89.5)	0.28	37.8%	33.3%	2.6	(0.24–26.9)	0.43	21.6%	33.3%	11.5	(2.43–54.5)	0.0021
Poor	24.3%	33.3%				39.2%	50.0%				37.8%	50.0%			
Poorest	59.5%	50.0%				23.0%	16.7%				40.5%	16.7%			

We merged “richest/very wealthy” and “rich/wealthy” with “middle” because of small numbers at these highest extremes of the distribution, to create a combined “middle & above” category in the three socio-economic status indexes, to facilitate data modelling. Analysis was done using logistic regression for trend, adjusted for clustering using robust standard errors method. Using the Benjamini and Hochberg method, only tests with a p-value below 0.0053 have a False Discovery Rate of <5%.

^**a**^ adjusted for age, sex, marital status and highest family education.

^**b**^ adjusted for age, marital status and highest family education.

^c^ adjusted for age, sex and highest family education.

^‡^To classify participants into young and old age groups, the median value of age was used as a cut-off point.

* Unmarried includes: single, divorced and widowed.

^†^ VI includes: moderate visual impairment, severe visual impairment and blindness. Cont = Controls; VI = Visual impairment.

### Self and Peer-Rated Wealth Indexes and Trichiasis

On both the self-rated and peer-rated scores, the households of trichiasis cases were rated poorer than controls (Table 3). This association persisted in both self-rated (OR = 3.99; 95%CI, 2.43–6.54; p<0.0001) and peer-rated (OR = 9.10; 95%CI, 4.79–17.27; p<0.0001) wealth measures after adjusting for marital status and highest family education. Compared to the controls, the trichiasis case households were more likely to be rated as poorest and poor rather than middle or affluent by themselves (OR = 3.74; 95%CI, 2.55–5.49; p<0.0001) and their peers (OR = 10.57; 95%CI, 6.42–17.41; p<0.0001) compared to the other households in their villages (Table 4). Using the 0 to 100 scale (poorest to richest), the mean self-rated scores for cases and controls were 34.1 v 49.1 (p<0.0001) and for peer-rated scores they were 27.5 v 50.3 (p<0.0001). The association of lower self-rated and peer-rated wealth with trichiasis persisted regardless of age, gender, marital status, and in people with normal visual acuity after adjusting for the matching variables and family education (Table 4).

### Reliability and Correlation of Economic Poverty Measures

The asset based socio-economic classification of households was found to be robust and produced similar ranking of households when the overall index was compared with the different subset indexes; the Spearman rank correlation coefficient ranged between 0.88 and 0.94. A Spearman rank correlation coefficient between asset index and self-rated wealth index, asset index and peer-rated wealth index, and self and peer-rated wealth indexes were 0.58, 0.70 and 0.63, respectively.

### Activity Participation and Trichiasis

Trichiasis cases were significantly less likely to participate in household, outdoor, agricultural and leisure activities, even after controlling for the presence of other health problems during the preceding month, (Table 5). However, the trichiasis cases were slightly more likely to participate in daily labouring and self-employment activities such as selling goods. These associations persisted in multivariable analysis after controlling for self reported health problems during the preceding month, except for leisure activities. In stratified analyses by vision, trichiasis cases with normal vision were significantly less likely to participate in processing of agricultural products and in productive outdoor activities such as fetching wood and travelling compared to controls with normal vision (Table 5).

**Table 5:** Associations between participation in an activity during the last week and case-control status; and stratified analyses by vision.

Activity	Cases		Controls		Adjusted Analysis ^c^			Normal Vision (N 320) ^d^					Visually Impaired (N 80) ^d^				
	n/200	(%)	n/200	(%)	OR	95% CI	P-value	Cases (n = 126)	Control(n = 194)	OR	(95% CI)	P-value	Cases (n = 74)	Control (n = 6)	OR	(95% CI)	P-value
***Productive household activities***																	
Cooking and cleaning dishes	166	(83.0)	168	(84.0)	0.63	(0.12–3.26)	0.58	84.1%	84.0%	0.94	(0.61–1.46)	0.7930	81.1%	83.3%	0.26	(0.03–2.49)	0.24
House cleaning	156	(78.0)	167	(83.5)	0.20	(0.05–0.77)	0.02	81.7%	83.5%	0.82	(0.53–1.29)	0.3961	71.65	83.3%	0.20	(0.02–2.34)	0.20
Washing clothing	99	(49.5)	129	(64.5)	0.49	(0.29–0.82)	0.006	55.6%	66.5%	0.59	(0.34–1.01)	0.0563	33.2%	0.0%	-	-	-
Looking after family member	129	(64.5)	137	(68.5)	0.99	(0.61–1.60)	0.97	72·2%	69.1%	1.18	(0.69–2.04)	0.5458	51.4%	50.0%	0.33	(0.06–1.79)	0.20
***Productive outdoor activities***																	
Shopping/Marketing	125	(62.5)	151	(75.5)	0.53	(0.31–0.90)	0.02	69.0%	76.8%	0.68	(0.38–1.23)	0.20	51.3%	33.3%	0.89	(0.18–4.39)	0.88
Fetching wood	78	(39.0)	153	(76.5)	0.09	(0.04–0.20)	<0.0001	42.1%	77.3%	0.21	(0.13–0.35)	<0.0001	33.9%	50.0%	0.23	(0.04–1.46)	0.12
Fetching water	151	(75.5)	170	(85.0)	0.38	(0.18–0.79)	0.01	79.4%	86.1%	0.55	(0.29–1.04)	0.07	68.9%	50.0%	0.89	(0.20–3.99)	0.88
Travelling	73	(36.5)	116	(58.0)	0.37	(0.23–0.60)	0.0001	34.9%	59.3%	0.36	(0.21–0.61)	0.0002	39.2%	16.7%	2.28	(0.23–22.1)	0.48
***Paid work***																	
Daily labouring	13	(6.5)	4	(2.0)	6.30	(0.79–50.95)	0.08	7.9%	2.1%	2.48	(0.87–7.11)	0.09	4.0%	0.0%	-	-	-
Self employment ^a^	38	(19.0)	25	(12.5)	2.08	(1.01–4.27)	0.05	24.6%	12.9%	2.39	(1.40–4.10)	0.002	9.5%	0.0%	-	-	-
***Agricultural activities***																	
Farming	93	(46.5)	118	(59.0)	0.55	(0.32–0.94)	0.03	48.4%	59.8%	0.72	(0.46–1.11)	0.14	43.2%	33.3%	1.28	(0.20–8.02)	0.79
Animal raring	130	(65.0)	165	(82.5)	0.23	(0.10–0.52)	0.0003	71.4%	83.5%	0.59	(0.37–0.96)	0.04	54.0%	50.0%	0.53	(0.13–2.21)	0.38
Processing agricultural products	95	(47.5)	160	(80.0)	0.16	(0.08–0.31)	<0.0001	50.8%	81.4%	0.24	(0.14–0.41)	<0.0001	41.9%	33.3%	0.74	(0.11–4.93)	0.75
***Leisure activities***																	
Social visits	141	(70.5)	149	(74.5)	0.88	(0.53–1.48)	0.64	69.8%	75.8%	0.88	(0.51–1.54)	0.67	71.6%	33.3%	3.46	(0.50–23.9)	0.21
Attending ceremonies	43	(21.5)	59	(29.5)	0.61	(0.33–1.11)	0.11	21.4%	30.4%	0.75	(0.44–1.25)	0.27	21.6%	0.0%	-	-	-
Attending social meetings	16	(8.0)	31	(15.5)	0.46	(0.20–1.04)	0.06	11.1%	16.0%	0.82	(0.44–1.53)	0.53	2.7%	0.0%	-	-	-
Relaxing activities ^b^	40	(20.0)	64	(32.0)	0.49	(0.29–0.83)	0.009	23.0%	31.4%	0.69	(0.40–1.20)	0.19	14.9%	50.0%	0.13	(0.02–0.90)	0.039

^a^ Selling goods

^b^ Listening to radio, Reading, Watching TV.

^c^ Conditional logistic regression adjusted for self reported health problem in the last month. Visual impairment included moderate visual impairment, severe visual impairment and blindness. A dashed line indicates that comparison is not possible.

^d^ Analysis was done using logistic regression adjusted for clustering using robust standard error methods and adjusted for age and self reported health problem. Odds ratios are relative to the controls. In the stratified analyses by vision, using the Benjamini and Hochberg method, only tests with a p-value below 0.002 have a False Discovery Rate of <5%.

After adjusting for the matching variables and self reported health problems, trichiasis cases were significantly more likely to report difficulty in performing all productive and leisure activities than the controls: >66% of the cases reported difficulty in all productive activities in contrast to <5% of controls (Table 6). Similarly, trichiasis cases were significantly more likely to report receiving assistance in doing all productive activities compared to controls. In contrast to other activities, higher proportions of trichiasis cases received assistance particularly in agricultural activities such as farming, animal husbandry and processing agricultural products (Table 6).

**Table 6:** Association between case-control status and having difficulty in doing an activity and receiving assistance to do it among those who have done the activity in the past week.

Activity	Difficulty with activity					Assisted with activity				
	Cases		Controls			Case		Control		
	n/N	(%)	n/N	(%)	P value	n/N	(%)	n/N	(%)	P value
***Productive household activities***										
Cooking and cleaning dishes	142/166	(85.5)	4/168	(2.4)	<0.0001	25/166	(15.1)	1/168	(0.6)	0.001
House cleaning	130/156	(83.3)	3/167	(1.8)	<0.0001	19/156	(12.2)	1/167	(0.6)	0.004
Washing clothing	66/99	(66.7)	1/129	(0.8)	<0.0001	13/99	(13.1)	0/129	(0.0)	-
Looking after family member	75/129	(58.10	1/137	(0.7)	<0.0001	27/129	(20.9)	1/137	(0.7)	0.0002
***Productive outdoor activities***										
Shopping /Marketing	96/125	(76.8)	4/151	(2.6)	<0.0001	4/125	(3.2)	1/151	(0.7)	0.38
Fetching wood	64/78	(82.1)	4/153	(2.6)	<0.0001	7/78	(9.0)	2/153	(1.3)	0.005
Fetching water	110/151	(72.8)	5/170	(2.9)	<0.0001	22/151	(14.6)	1/170	(0.6)	0.002
Travelling	62/73	(84.9)	2/116	(1.7)	<0.0001	7/73	(9.6)	1/116	(0.9)	0.05
***Paid work***										
Daily labouring	10/13	(76.9)	0/4	(0.0)	-	1/13	(7.7)	0/4	(0.0)	-
Self employment	27/38	(71.0)	1/25	(4.0)	0.0001	4/38	(10.5)	0/25	(0.0)	-
***Agricultural activities***										
Farming	81/93	(87.1)	0/118	(0.0)	-	27/93	(29.0)	2/118	(1.7)	<0.0001
Animal raring	98/130	(75.4)	5/165	(3.0)	<0.0001	63/130	(48.5)	20/165	12.1	<0.0001
Processing agricultural products	80/95	(84.2)	1/160	(0.6)	<0.0001	12/95	(12.6)	0/160	(0.0)	-
***Leisure activities***										
Family/Social visits	57/141	(40.4)	2/149	(1.3)	<0.0001	2/141	(1.4)	0/149	(0.0)	-
Attending ceremonies	26/43	(60.5)	0/59	(0.0)	-	1/43	(2.3)	0/59	(0.0)	-
Attending social meetings	8/16	(50.0)	0/31	(0.0)	-	1/16	(6.2)	0/31	(0.0)	-
Relaxing activities ^a^	12/40	(30.0)	2/64	(3.1)	0.003	0/40	(0.0)	1/64	(1.6)	-
***Activities of daily living***	79/200	(39.5)	2/200	(1.0)	<0.0001	6/200	(3.0)	0/200	(0.0)	-

The denominators are the number of participants who did the activity in the past week. Analysis was done using logistic regression adjusted for clustering using robust standard errors method and adjusted for the matching variables (age & sex) and self reported health problem. Only P-values are presented as the cell sizes of the majority were too small for calculation of odds ratio. Using the Benjamini and Hochberg method, only tests with a p-value below 0.005 have a False Discovery Rate of <5%. A dashed line indicates that comparison is not possible.

### Factors Associated with Asset Based Household Economic Poverty in Trichiasis Cases

In a univariable analysis (Table 7), being a household head with trichiasis had a strong association with economic poverty (OR = 3.29; 95%CI, 1.89–5.75; p<0.0001) while visual impairment had a borderline association (OR = 1.71; 95%CI, 0.98–2.97; p = 0.058). Not having a marriage partner (OR = 9.41; 95%CI, 4.16–21.31; p<0.0001), no family member with formal education (OR = 4.95; 95%CI, 1.73–14.16; p = 0.0028) and a main family job of daily labouring (OR = 19.64; 95%CI, 2.32–166.49; p = 0.0063) as opposed to farming were independently associated with economic poverty (Table 7). Families in which there were more people of a productive age were less likely to be poor than their counterparts (OR = 0.32; 95%CI, 0.16–0.60; p = 0.0005) (Table 7). In a multivariable analyses, participating in animal husbandry (OR = 0.05; 95%CI, 0.02–0.12; p<0.0001) and agricultural product processing (OR = 0.50; 95%CI, 0.27–0.91; p = 0·024) activities were independently associated with wealthier households while house cleaning (OR = 2.05; 95%CI, 1.03–4.08; p = 0.042) and self employment (OR = 2.77; 95%CI, 1.25–6.18; p = 0.012) activities were associated with poorer households.

**Table 7:** Univariable and multivariable ordinal logistic regression for household economic poverty among the 200 trichiasis cases only.

Variable	OR	95% CI	p-value
***Univariable analysis***			
Trichiasis case is household head	3.29	(1.89–5.75)	<0.0001
Marital status, being single/widowed/divorced	12.14	(5.71–25.82)	<0.0001
Productive age family members ≥3	0.17	(0.09–0.30)	<0.0001
Highest family education, No formal education	8.09	(3.24–20.20)	<0.0001
Highest family job			
Farmer (reference)	1	-	-
Self employed/employed	7.00	(1.97–24.81)	0.003
Daily Labourer	20.11	(2.60–155.50)	0.004
Trichiasis severity (Major TT)	0.93	(0.55–1.57)	0.78
Visual impairment	1.71	(0.98–2.97)	0.06
***Multivariable logistic regression***			
Marital status, Single/widowed/divorced	9.41	(4.16–21.31)	<0.0001
Productive age family members ≥3	0.32	(0.16–0.60)	0.0005
Highest family education, No formal education	4.95	(1.73–14.16)	0.003
Highest family job			
Farmer (reference)	1	-	-
Self employed/employed	6.63	(1.62–27.11)	0.008
Daily Labourer	19.64	(2.32–166.49)	0.006

Analysed based on the classification of participants and households into quintiles (richest to poorest) using the overall asset index. Ordinal logistic regression was used to identify correlates of asset based socio-economic status (ordered categorical variable) in a univariable and multivariable analysis. Variables that were associated with the outcome on univariable analyses at a level of p<0.05 were included in the multivariable analysis and then those with p<0.2 were retained in the final model after likelihood ratio-test.

## Discussion

Poverty is a complex multidimensional issue that encompasses not only deprivation of material possessions but also wider issues such as nutrition, health and education [32,33]. Many different approaches have been taken to measuring “poverty”, both in absolute and relative terms [34]. In general, these involve a survey methodology to capture estimates of income or consumption and methods that take into account broader issues of health and education such as the Multidimensional Poverty Index [34].

According to the 2011 World Bank estimates, 29.6% (Urban, 25.7%; Rural, 30.4%) of Ethiopians live below the national absolute poverty line (defined as 3781 Birr) and 30.7% live on less than US$1.25 PPP (purchasing power parity) a day [35]. Using asset indicators, the World Bank defines a household as being deprived “when none of these assets are owned by the household: fridge, phone, radio, TV, bicycle, jewelry, or vehicle” [35]. According to these criteria, 53% of rural households in Ethiopia were in deprivation in 2011. However, these are narrowly defined assets and most of these would not be commonly found in a rural Ethiopian community, irrespective to the level of wealth [35].

In this study we compared individuals with trichiasis to matched controls from within the same communities in Amhara Region, Ethiopia using three different measures of relative poverty: Asset Index, Self-Rated Wealth Index and Peer-Rated Wealth Index. These measures allow us to understand whether people with TT were relatively poorer than their neighbours, even within these very poor communities. We performed a PCA of household assets to stratify the participants into economic groupings. The variance explained by the first principle component was similar to the range reported in other similar studies (between 11% and 27%) [19,20,27,36]. The asset index used in this study is probably a reasonable proxy for consumption expenditure as we collected data on a sufficiently broad set of asset indicators that are capable of capturing living standards and wealth inequalities based on local values [37].

### Participant and Household Characteristics

The age distribution, gender profile and literacy status of the trichiasis cases in this study were comparable with those reported in our earlier studies in Ethiopia as well as other studies of trichiasis patients elsewhere in Sub-Saharan Africa [31,38–40]. This suggests that the results are probably generalizable for this region of Ethiopia at least. The households of trichiasis cases were significantly less well off than controls in terms of ownership of almost all asset indicators measured. Consistent with the literature, trichiasis cases had significantly smaller and more crowded households [6,41]. Cases had less latrine access and more kept their cattle within the house, which is consistent with observations that active trachoma is associated with poor sanitation access [41–43]. These differences reflect a gap in the implementation of the “E” component of the SAFE strategy, which needs on-going emphasis in this region.

### Trachoma and Poverty

We have found clear evidence from each measure that even within trachoma-endemic communities individuals and households affected by trichiasis are significantly economically poorer than those that are not. Within endemic communities some individuals or families appear to be more severely affected by the disease and develop sight-threatening complications. This raises the important question of whether the association between poverty and trichiasis arises from a general state of impoverishment or whether there are a number of critical factors that primarily drive the relationship that might be amenable to focused intervention. The data we present here suggest that the relationship between poverty and trachoma could possibly be bidirectional.

Poverty may contribute to trachoma. This study provides evidence that even within superficially homogeneous endemic communities relative poverty plays a major part in the vulnerability of families to scarring disease. Firstly, trichiasis cases were more likely than the controls to come from households where the main family job is daily labouring and from families with no or lower formal education. Both of these factors have a major influence on income and health awareness, which in turn increase the vulnerability of the family to trachoma. Consistent with this, studies from Malawi, Tanzania and Ethiopia identified that children from lower socio-economic households had a higher prevalence of active trachoma than their counterparts indicating an association between poverty and active trachoma [10,44,45]. Secondly, previously described risk factor associations for active trachoma such as crowding and poor access to latrine, characterised the households of the trichiasis cases in this study. Such conditions are believed to promote the transmission of *Chlamydia trachomatis* within endemic communities, sustaining higher prevalence levels. Poorer households and communities may be less likely to have either the resources or the awareness to access treatment and sustain a sufficiently hygienic environment to control trachoma [8,17,46,47]. Households with higher income were more likely to have a latrine than their counterparts in a study conducted in the same area [48].

Trachoma may also contribute to poverty. Poor health frequently results in loss of productivity through disability and diversion of resources [11]. Trichiasis and its associated visual impairment probably lead to a loss of income, exacerbating pre-existing poverty in a “vicious cycle” [12,13]. Previously healthy and productive adults can be rendered dependent on others, unable to work or fully care for themselves due to pain, photophobia or visual impairment [13]. We found clear evidence of reduced activity and participation among trichiasis cases. Trichiasis cases were less likely than the controls to participate in productive household activities, outdoor activities (shopping/marketing, fetching wood and water) and agricultural activities (farming, animal husbandry and processing agricultural products). The stratified analysis found trichiasis cases with normal vision are less likely to participate in outdoor and agricultural activities than controls. This is consistent with a study of Tanzanian women with trichiasis without visual impairment, who had a degree of functional limitation which was comparable to those with visual impairment [14]. We found evidence that households with fewer economically productive adults and where the family head had trichiasis tended to be poorer. Conversely, households where trichiasis cases participated in agricultural activities were better off. Even where the trichiasis cases were undertaking specific activities, they reported much more difficulty and greater need for assistance than the controls. Similarly in another study, trichiasis cases reported difficulty in performing day-to-day farming activities [49]. These observations all point towards households with someone with trichiasis being under greater financial strains through reduced income contribution and greater needs and dependence of the person with trichiasis. The burden of disability caused by trachoma has been estimated between 171,000 and 1.3 million DALYs, with economic losses of 5–8 billion USD/year [4,12,13]. The economic loss from trichiasis alone due to lost productivity was estimated to be 3 billion USD/year [12,13].

### Study Strengths

This study comprehensively assesses the relationship between trachoma and economic poverty using four different measures, with a robust process to select suitable community controls. The asset index quantifies the long-term economic welfare of trachoma affected communities, which is important as trachoma and its sequelae are probably related to long-term SES [19,20]. The asset index has the practical advantage that it is much less affected by recall or measurement bias during data collection [19]. Most of the housing characteristics, utilities and durable assets were collected through direct observation minimising miss-measurement. Broad ranges of asset data were collected increasing the power of the study in the following ways. Clumping and truncation, potential problems that can arise with PCA of asset data and compromise its suitability for defining socio-economic strata, did not occur when all asset indices were combined into a single index. This indicates that the data from this study is sufficient to measure economic status and effectively infer inequality between different socio-economic strata and that in this region assessment of economic status by asset measurement requires a wider pool of parameters, particularly including agricultural assets. Encouragingly, the asset based poverty measure was moderately and strongly correlated with the self-rated and peer-rated wealth measures.

### Limitations of the Study

Poverty is a complex multidimensional problem with many causes and manifestations. Therefore there are many ways in which poverty can be measured. Here we only examined the economic aspect using relative measures such as low asset ownership. We use the first principal component (PC1) to measure socio-economic status. However, there is no clear description of the number of principal components to use and often the factor scores derived from the other principal components are difficult to interpret [27]. Despite the comparability of the amount of variance explained by PC1 with other studies, there is uncertainty whether the first component alone sufficiently explains all the pertinent variation. Asset scores are usually developed to be locally relevant, to allow ranking of people within the same community with respect to poverty. Unfortunately, socio-economic classifications based on asset ownership quintiles measure relative poverty within a given context and face the limitation of lacking international comparability. Therefore, between region or country comparison of SES should be done with caution [28]. We did not collect consumption or expenditure data, and so were not able to assess absolute poverty levels.

Although a community based screening method was used to identify trichiasis cases, it is possible that some cases might have been missed, which could potentially introduce non-response bias. Similarly, it is possible that some potential controls were not listed by the sub-village administrators. Self and peer-rated wealth are subjective measures, which might have suffered from the tendency to favour ranking households in the middle of the distribution. The activity participation data relied on the participant’s recall ability on what s/he had done in the last week. Finally, our results suggest that a bidirectional relationship may possibly exist between trachoma and poverty. However, the authors recognise that inference about causality is speculative as it is not possible to draw firm conclusions from a cross-sectional observational study such as this.

### Conclusions

In this study we found a clear association between trichiasis and household economic poverty by all three economic measures. Trichiasis cases were more likely to have economically poor households and less likely to participate in productive activities regardless of visual impairment, more likely to report difficulty in performing productive activities and more likely to need assistance in performing activities than controls. These suggest that the causative relationships between poverty and trachoma may possibly involve bidirectional interaction: poor households are more affected by trachoma and the scarring sequelae of trachoma and trichiasis reduces productivity even prior to the development of visual impairment, which might lead to additional poverty.

These data are anticipated to be useful in advocacy and to support programme leaders and funders to secure resources to promote trachoma prevention linked to socio-economic development in trachoma-endemic communities. Implementation of the full SAFE strategy in the context of general improvements might lead to a virtuous cycle of improving health and wealth. Trachoma is a good proxy of inequality within communities and it could be used to target and evaluate interventions for health and poverty alleviation. Measuring the effect of trichiasis surgery on household economic poverty through longitudinal studies would provide an indication of the relative contribution of trichiasis to poverty, as improved health potentially leads to improved productivity and income.

## Supporting Information

S1 Checklist STROBE Checklist. (DOCX)

S1 Table Summary statistics and principal component factor scores for asset variables used in the Principal Component Analysis (PCA). (PDF)
