# Supplementary material for: Trachoma and Relative Poverty: A Case-Control Study
Source: PLoS Negl Trop Dis. 2015 Nov 23;9(11):e0004228. doi: 10.1371/journal.pntd.0004228 (PMC4657919; doi:10.1371/journal.pntd.0004228)
Supplement: S1 Table — (PDF) [file pntd.0004228.s002.pdf]

S1 Table: Summary statistics and principal component factor scores for asset variables used in the Principal Component Analysis (PCA).

Variables	Summary Statistics		Factor score for PC1				A change from 0 to 1	Means of all indicators asset index by quintile				
	Mean	*SD*	*Housing*	*Durable assets*	*Agricultural assets*	*All*	*Fi/Si*	*Poorest*	*poor*	*Middle*	*Rich*	*Richest*
Housing characteristics and utilities												
Own the house	0·930	0·255	0·006			0·161	0·635	0·804	1·000	1·000	1·000	1·000
Number of rooms	1·383	0·730	0·441			0·154	0·208	1·182	1·286	1·281	1·617	1·980
Roof made of metal	0·925	0·264	0·315			0·143	0·538	0·804	0·978	1·000	1·000	1·000
Number of metal roof sheets	50·58	28·48	0·435			0·320	0·011	27·20	51·99	58·84	64·57	89·47
Cattle dwelling within the main house	0·540	0·499	-0·365			-0·024	-0·050	0·441	0·802	0·789	0·383	0·245
Cattle dwelling separate to the main house	0·240	0·428	0·424			0·242	0·570	0·028	0·110	0·175	0·600	0·735
Has latrine	0·668	0·472	0·178			0·078	0·161	0·503	0·769	0·789	0·700	0·776
Kitchen separate from the main house	0·380	0·486	0·256			0·035	0·072	0·378	0·275	0·368	0·400	0·571
Access to electric	0·175	0·380	0·216			-0·096	-0·261	0·329	0·099	0·053	0·033	0·184
Own other house/s	0·088	0·283	0·245			0·046	0·152	0·056	0·143	0·000	0·100	0·163
Ownership of durable assets												
Phone	0·243	0·429		0·562		0·076	0·175	0·224	0·154	0·175	0·283	0·490
Radio/tape recorder	0·290	0·454		0·509		0·180	0·394	0·133	0·209	0·228	0·500	0·714
Number of furniture	1·243	0·892		0·534		0·141	1·279	1·021	1·176	1·228	1·383	1·857
Cart	0·068	0·251		0·373		0·123	0·498	0·021	0·011	0·035	0·150	0·245
Agricultural assets												
Mango Tree	0·078	0·268			0·192	0·152	0·582	0·007	0·044	0·035	0·117	0·347

Guava Tree	0·063	0·242			0·170	0·138	0·583	0·000	0·033	0·070	0·083	0·265
Lemon Tree	0·083	0·275			0·213	0·170	0·629	0·014	0·011	0·053	0·150	0·367
Banana Tree	0·090	0·287			0·213	0·178	0·620	0·007	0·011	0·105	0·183	0·347
Buckthorn trees	0·685	0·465			0·284	0·173	0·503	0·343	0·758	0·895	0·983	0·939
Coffee land	0·093	0·290			0·143	0·107	0·362	0·028	0·066	0·070	0·167	0·265
Equaliptous tree land	0·535	0·499			0·315	0·247	0·499	0·112	0·626	0·877	0·767	0·918
Teff and other cereals, land in Hectares	0·961	0·713			0·375	0·301	0·426	0·417	0·901	1·237	1·342	1·872
All land in Hectares	1·032	0·751			0·392	0·319	0·429	0·447	0·933	1·292	1·463	2·089
Number of oxen and cows	3·605	3·267			0·369	0·324	0·099	0·930	3·286	4·772	5·267	8·612
Number of horses and mules	0·540	0·797			0·289	0·258	0·327	0·042	0·341	0·895	0·933	1·469
Number of sheep and goats	1·670	2·518			0·274	0·219	0·087	0·427	1·286	1·789	2·833	4·449
Number of chickens	2·889	4·263			0·235	0·190	0·045	0·853	2·582	3·737	4·033	7·000
Household took government loan	0·125	0·331				-0·038	-0·112	0·140	0·231	0·070	0·050	0·041
**Overall index**	0·000	2·392										
Eigenvalues associated with PC1			2·54	1·88	4·31	5·72						
Share of variance associated with PC1			25%	41%	33%	21%						
Number of variables used			10	4	13	28						
KMO sample adequacy test			0·56	0·65	0·79	0·79						

This table shows the summary statistics of the all asset indicators used in the PCA in the first two columns. In the next 4 columns, the factor score of the first principal component for each three indices and when all indicators are combined as a single indicator is presented. The factor scores are the weight assigned to each asset indicator, normalised by its mean and standard deviation, in the linear combination of the variable that constitutes the first principal component. The 7th column shows the factor score of each indicator divided by its standard deviation. This shows how much owning an asset contributes to the index compared to not owning in dummy variables. A positive value reflects an increase and a negative value a reduction in the asset score. The last five columns show the mean value of the asset ownership across the five socio-economic groupings of case and controls together classified based on the "cut points" of the controls' *first factor score quintiles. In most of the asset indicators with positive and negative factor scores, the mean asset ownership of assets increased and decreased respectively from poor to rich households.*
